# The physiological cost of male-biased parasitism in a nearly monomorphic mammal

**DOI:** 10.1186/s13071-017-2060-5

**Published:** 2017-04-21

**Authors:** Arturo Oliver-Guimerá, Carlos Martínez-Carrasco, Asta Tvarijonaviciute, María Rocío Ruiz de Ybáñez, Jordi Martínez-Guijosa, Jorge Ramón López-Olvera, Xavier Fernández-Aguilar, Andreu Colom-Cadena, Gregorio Mentaberre, Roser Velarde, Diana Gassó, Mathieu Garel, Luca Rossi, Santiago Lavín, Emmanuel Serrano

**Affiliations:** 1 Servei d’Ecopatologia de Fauna Salvatge (SEFaS), Wildlife Health Service, Departament de Medicina i Cirurgia Animal, Universitat Autònoma de Barcelona, E-08193 Bellaterra, Spain; 2 Departamento de Sanidad Animal, Facultad de Veterinaria, Campus de Excelencia Internacional Regional “Campus Mare Nostrum”, Universidad de Murcia, E-30100 Murcia, Spain; 3 Departamento de Medicina y Cirugía Animal, Facultad de Veterinaria, Campus de Excelencia Internacional Regional “Campus Mare Nostrum”, Universidad de Murcia, Campus de Espinardo, E-30100 Espinardo Murcia, Spain; 4 Instituto de Investigación en Recursos Cinegéticos IREC (CSIC-UCLM-JCCM), E-13071 Ciudad Real, Spain; 5 Unité Faune de Montagne, Office National de la Chasse et de la Faune Sauvage (ONCFS), 34990 Juvignac, France; 6 Dipartimento di Scienze Veterinarie, Università degli Studi di Torino, Grugliasco, 10095 Torino, Italy; 7 Departamento de Biologia & CESAM, Universidade de Aveiro, 3810-193 Aveiro, Portugal

**Keywords:** Gastrointestinal nematodes, Lung nematodes, Kidney fat reserves, Oxidant/antioxidant status, *Rupicapra pyrenaica pyrenaica*

## Abstract

**Background:**

Even though male-biased parasitism is common in mammals, little effort has been made to evaluate whether higher parasitic burden in males results in an extra biological cost, and thus a decrease in fitness. Body condition impairment and the augmentation of oxidative stress can be used as indicators of the cost of parasite infections. Here, we examined relationships between gastrointestinal and respiratory helminths, body condition and oxidative stress markers (glutathione peroxidase, paraoxonase-1) in 28 Pyrenean chamois (*Rupicapra p. pyrenaica*) sampled in autumn.

**Results:**

Only male chamois showed a reduction in body condition and higher oxidative stress due to parasite infection, likely because of the extremely high parasite burdens observed in males.

**Conclusions:**

This study made evident a disparity in the physiological cost of multiple parasitism between sexes in a wild mammal, mainly due to parasitic richness. Because of the similar life expectancy in male and female chamois, we suggest that males may have developed natural mechanisms to compensate for higher parasite loads during the rut.

## Background

Sex-based differences in exposure, susceptibility [1] and tolerance lead to differences in parasite prevalence [2], intensity [3], and pathology [4] in a broad range of vertebrate species. Males are often more infected than females as observed in birds [5], rodents, bats [6], ungulates [1, 3] and humans [4]. This male-biased parasitism has been linked to both hormonal and behavioural differences. Generally, males invest more energy than females in the development of testosterone-mediated traits, such as secondary sexual traits or courtship displays. Although testosterone is necessary for developing of secondary sex characteristics in males, high testosterone levels have been linked to a suppressed immune system [4, 7] and increased parasitism. Male-biased infection is common in sexually dimorphic and polygynous species [8], and might reduce male fitness. However, in sexually dimorphic species it might be difficult to separate the physiological costs of parasitism from the costs of the production and maintenance of sexually-selected traits [9]. Monomorphic species, in which males and females are morphologically similar, provide ideal systems for examining the physiological costs of male-biased parasitism.

Here we compare the physiological costs of male-biased parasitism in a nearly monomorphic ungulate (only small differences in body mass can be observed in autumn), the Pyrenean chamois (*Rupicapra pyrenaica pyrenaica*). Found in the mountains of southern Europe, chamois are medium-sized polygynous ungulates [10]. Males are more heavily parasitized [3], however, life expectancy is similar between the sexes [10].

We assess the physiological costs of infection using both general indicators of nutritional status, such as indicators of body condition (e.g. kidney fat stores, KFs [11]) and insulin-like growth factor 1 (IGF1), and oxidative stress (OS) markers, such as paraoxonase 1 (PON1), total antioxidant capacity (TAC) and glutathione peroxidase (GPX). Oxidative stress originates from an imbalance between the production of reactive oxygen or nitrogen species, and the capacity of the organism to compensate for their detrimental effects or destroy these harmful products [12]. As result, OS biomarkers are widely used to measure infection damage in veterinary sciences [13] and conservation physiology [14] and are considered good animal health indicators of animal health. The proper assessment of oxidative status must consider not only the concentration of antioxidants (e.g. antioxidant vitamins or endogenous enzymes), but also oxidative damage. For example, an elevation in endogenous antioxidants with stable oxidative damage concentrations suggests that individuals are successfully dealing with oxidative stress. In contrast, the depletion of antioxidant substances and stable oxidative conditions suggest oxidative damage.

In this work, we assess the endoparasite (gut and respiratory helminths) and physiological status (oxidative stress and nutritional status) of 28 adult Pyrenean chamois. Using these data, we tested whether, for a given parasite load, males have a higher physiological cost than females. Because male chamois are prone to suffer from malnourishment due to rut-induced hypophagia (mainly territorial individuals, see [15]), we expect higher physiological costs (i.e. lower nutritional status and higher oxidative stress) in males than in females for a given parasite burden. On the other hand, because both female and male chamois have similar life expectancies we expect that males may have developed physiological adaptations to compensate for the damage caused by parasites during the rutting period.

## Methods

### Chamois sampling

The respiratory and gastrointestinal systems of 28 adult Pyrenean chamois, 17 females (mean age 9.2 years, range 3–21) and 11 males (mean age 7.5 years, range 3–12) from the Freser-Setcases National Game Reserve (Catalan Pyrenees, northeast Spain) were collected. Animals were hunter-harvested from October to December in 2012 and 2013, coinciding with the rut and a period of poor food quality [16]. Once the animal was dead, heart puncture was performed to obtain blood samples, which were allowed to clot at room temperature and centrifuged at 1800 G. Serum was transported at 4 °C and frozen at −80 °C until analysis. Abdominal and thoracic viscera were extracted and kidneys were stored with their fat. The organs were placed in labelled plastic bags and transported in a refrigerated box at 4 °C to the laboratory, where the material was stored at −20 °C until parasitological examination. Kidney fat reserves were used as a proxy for body condition in sampled chamois [11].

### Parasitological data

Adult nematodes were recovered from the lungs, preserved in 70% ethanol and later mounted in lactophenol for identification [17]. Lung larvae were obtained using the Baermann method on 30 g of lung parenchyma. Liquid was collected and centrifuged (800× *g*). Larvae were identified under a microscope using a Favatti chamber [18].

### Biochemical analysis

PON-1 activity was determined using p-nitrophenyl acetate as a substrate [19]. Serum paraoxonase (PON1) is an enzyme able to neutralise lipid-peroxides (oxidised lipids) that cause inflammation among other organic and inorganic substrates. The TAC (ability of antioxidants to clear harmful free radicals) was measured using a method based on 2.2′-azinobis-(3-ethylbenzothiazoline-6-sulfonate) decolorization by antioxidants [20]. TAC considers the cumulative action of all the antioxidants present in body fluids. Thus, a low TAC can be indicative of oxidative stress or increased susceptibility to oxidative damage. GPX was measured according to a previously described method [21]. The main biological role of GPX is to reduce lipid hydroperoxides to their corresponding alcohols and free hydrogen peroxide to water. All three analyses were measured in serum with an automated biochemistry analyser (Olympus AU600; Olympus Europe GmbH, Hamburg, Germany). IGF-1 is a hormone that plays an important role in the growth of juveniles and anabolism in adults and it was analysed with an automated solid-phase, enzyme-labelled chemiluminescent immunometric assay (Immulite System, Siemens Health Diagnostics, Deerfield, IL, USA). All analyses showed inter- and intra-assay coefficients of variation below 15%, and recovery ranged between 80 and 120% in all cases.

### Statistical analyses

The lung nematode load obtained in the present study was analysed in combination with the data on gastrointestinal parasite load recorded for the same individuals and previously published by Martínez-Guijosa et al. [3]. The partial least squares (PLS) regression method was used to assess the relationships between parasitism and health in female and male chamois. As a response variable, we defined a “Cost of infection block”, which included markers of oxidative stress (GPX, PON1), antioxidant capacity (TAC), and the nutritional status of individuals (IGF-1 and KFs). The endoparasite infection block (set of predictor variables) was represented by the intensity of each parasite species (number of parasite individuals of each species), the total intensity (including all species) and the species richness of lung and gastrointestinal parasites. The PLS regression is suitable when sample size is low relative to the number of variables and when collinearities occur among predictor variables [22]. The loads, weights (i.e. relative contribution of each variable to the derived factors), and cross-correlations (e.g. the correlation between each X variable and the whole Y component, i.e. PLS’ scores, and *vice versa*) were also estimated. Finally, the Stone-Geisser test (Q^2^) at a threshold of 0.0975 was used to assess the fit of the PLS model. The Q^2^ statistic can be viewed as a jackknife version of the R^2^ statistic. Analyses were performed with the “plspm” package [23] in R, version 3.3.2 [24].

## Results

Similar to gastrointestinal parasites [3], prevalence and intensity of lung nematodes and their larvae were higher in males than in females (Table 1). Regarding the health indicators, however, only the PON1 concentration was significantly different between sexes after a Bonferroni correction (*t*_ (24.8)_ = 5.03, *P*-value < 0.001, Table 2).

**Table 1:** Prevalence at 95% confidence interval (95% CI), mean and median intensity and its range (min-max) of adult and larval respiratory helminths in Pyrenean chamois (17 females and 11 males) from the Freser-Setcases National Game Reserve, northeast Spain

	Prevalence (%) (95% CI)		Mean intensity		Median intensity		Intensity range	
	Male	Female	Male	Female	Male	Female	Male	Female
Adult parasites								
*Protostrongylus rufescens boevi*	9.1 (0–26.1)	0 (0–0)	3	0	0	0	0–3	0
*Protostrongylus rufescens rufescens*	90.9 (73.9–100)	43.8 (19.4–68.1)	62.7	2	12	0	0–241	0–5
*Protostrongylus rupicaprae*	90.9 (73.9–100)	62.5 (38.8–86.2)	89.3	4.1	36	2	0–343	0–10
*Spiculocaulus austriacus*	100 (100–100)	31.3 (8.5–54.0)	52.5	2.4	17	0	1–311	0–4
Total	100 (100–100)	81.3 (62.1–100)	191	5.2	70	4	1–845	0–12
Larval parasites								
*Muellerius* spp*.*	63.6 (35.2–92.1)	56.3 (31.9–80.6)	303.8	70.4	4	4	0–1669	0–241
*Neostrongylus linearis*	100 (100–100)	75.0 (53.8–96.2)	128	165.9	23	13	1–594	0–701
*Protostrongylus* spp.	100 (100–100)	68.8 (46.0–91.5)	1256.4	126.8	107	7	1–10,609	0–650
Total	100 (100–100)	100 (100–100)	1577.7	157.7	129	49	2–10,994	1–1071

**Table 2:** Mean ± standard deviation, minimum and maximum values of selected physiological indicators to assess the health status of Pyrenean chamois (17 females and 11 males) in Freser-Setcases National Game Reserve, northeast Spain

Health status indicators	Male	Female
Oxidant status		
PON1 (units/l)	8.57 ± 2.39 (5.81–12.63)	14.68 ± 3.91 (5.28–19.33)
GPX (units/mg)	546.54 ± 385.15 (224–1532)	549.75 ± 300.95 (192–1200)
Antioxidant status		
TAC (mmol/l)	0.29 ± 0.13 (0.11–0.48)	0.35 ± 0.18 (0.02–0.68)
Nutritional status		
IGF-1 (ng/ml)	71.91 ± 53.26 (17.11–211.01)	60.36 ± 28.69 (13.71–137.01)
Kidney weight (g)	39.01 ± 5.61 (28.52–46.51)	35.36 ± 6.08 (25.30–50.91)
Kidney fat weight (g)	53.25 ± 12.53 (33.55–76.80)	61.65 ± 18.35 (34.55–90.52)

*Abbreviations*: *PON1* serum paraoxonase 1; *GPX* glutathione peroxidase; *TAC* total antioxidant capacity; *IGF-1* insulin-like growth factor 1

**Fig. 1:**
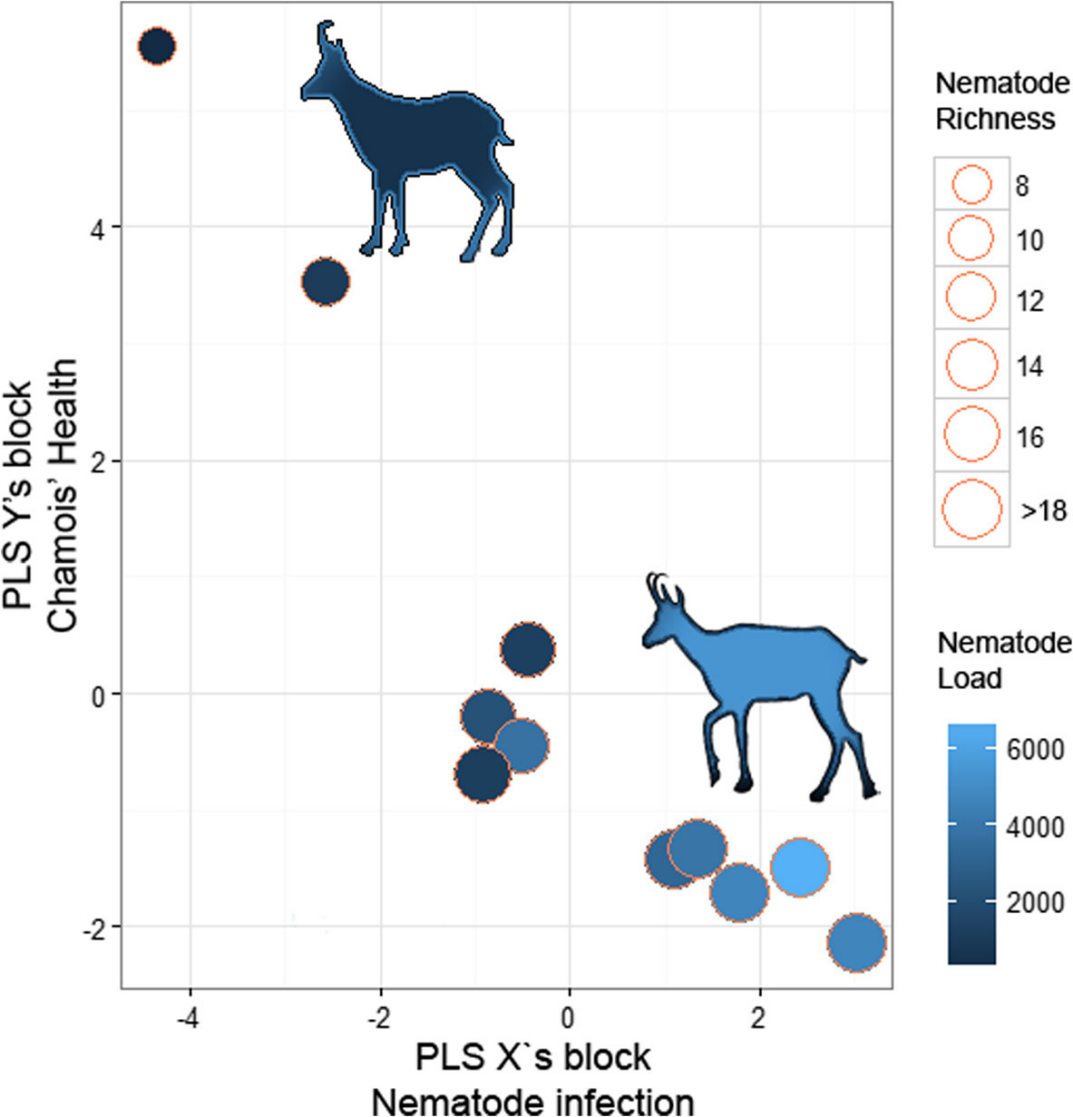
Bubble chart showing the relationships between PLS X’s component, representing the gut and lung nematode community, and the PLS Y’s component representing the health status in eleven male chamois from the Catalan Pyrenees, northeast Spain. The nematode community block was mainly represented by gastrointestinal and lung nematode richness and by gastrointestinal nematode intensity of infection, whereas the health block was represented by glutathione peroxidase (GPX) and paraoxonase-1 (PON1) concentrations, and by body condition. The initial set of variables representing each block is provided in Table 3. Bubble size indicates total (gut and lung) nematode richness ranging from 0 to 19 nematode species. Bubble colour indicates nematode burden. Chamois silhouettes represent a male chamois with low parasite load and hence in good condition and with a good antioxidant response (*top left*) and a highly parasitized male chamois in poorer condition and lower antioxidant response (*bottom right*)

After a preliminary analysis, we decided to exclude the IGF-1 and TAC from the Y block due to their low contribution (<2%) to the health block. Along the same lines, predictor variables with little or no influence on the X’s component (e.g. burdens of specific parasite species, such as cestodes or *Cooperia oncophora*) were also discarded to simplify our PLS model. In female chamois, the health block showed no correlation with parasite infection (Q2 = -0.57). In contrast, the male group analysis revealed a marked correlation (5.7 times greater than females; Q2 = 0.22), where 57.1% of the observed variability of chamois health status was accounted for by parasite infections. As a result, gastrointestinal helminth richness, respiratory nematode richness and total gastrointestinal intensity, in decreasing order of importance, had the greatest negative effect on the Y block (Table 3, Fig. 1). In the case of females, only 1.9% of the health status was related to parasite infections.

**Table 3:** Predictor weights of the most parsimonious Partial Least Squares (PLS) regression model explaining the physiological cost of parasitism in male chamois from the Freser-Setcases National Game Reserve, northeast Spain

PLS blocks	Predictor variables	Loads	Weights	Percent	Cross-correlation
X	**GI richness**	-0.41	-0.54	29.25	-0.99
	**LN richness**	-0.37	-0.46	21.41	-0.85
	**Total GI intensity**	-0.37	-0.39	15.04	-0.71
	***Nematodirus*** ** spp.**	-0.31	-0.32	10.51	-0.59
	*Marshallagia* spp.	-0.19	-0.25	6.49	-0.47
	Total LN intensity	-0.27	-0.25	6.12	-0.45
	*P. r. rufescens*	-0.28	-0.23	5.48	-0.43
	*S. austriacus*	-0.27	-0.23	5.41	-0.43
	LN larval richness	-0.08	-0.05	0.29	-0.10
Y	PON1	0.44	–	81.81	0.83
	GPX	0.37	–	88.34	0.72
	Body condition	0.35	–	77.98	0.71

Predictor variables explaining more than 10% in the response block are shown in bold type. In the PLS Y’s block the Y U correlation has been shown as a proxy for variable importance

*Abbreviations*: GI richness, number of gastrointestinal helminth species; LN richness, number of lung nematode species; Total GI intensity, number of adult nematodes in the gastrointestinal tract; Total LN intensity, number of adult nematodes in the lung; LN larval richness, number of larvae nematode species in the lung

## Discussion

Chamois with low parasitic intensities and richness showed higher PON1 and GPX concentrations (for a given TAC concentration) and were in better body condition than their more parasitized counterparts. On the other hand and line with previous work [25], we found that multiple infections have a greater physiological cost than single-parasite infections. However, interpreting sex differences in the physiological cost of parasitism is more challenging for several reasons, the first being the fact that females were by far less parasitized than males.

In males, the poor body condition and reduced antioxidant defences in highly parasitized individual chamois suggest an overload in the host’s capacity to overcome the infestation. Lower parasite burdens and richness show higher levels of antioxidant biomarkers (PON1 and GPX), resulting from up-regulation of endogenous antioxidants or mobilization of exogenous antioxidants, along with better body condition. Nonetheless, both sexes have similar lifespan, suggesting that male chamois employ compensatory mechanisms to deal with the extra-cost of parasite infections during the rut season. Females, however, do not seem to need these physiological compensations at this time of the year.

Many parasite infections in wildlife tend to be relatively benign, remaining subclinical until the host experiences additional stress [26]. Thus, many of the pathogenic mechanisms would be driven by the host’s own immune system, which is also energetically demanding. In these cases, when the cost of maintaining resistance outweighs the benefits, it has been postulated that tolerance is preferred [27]. This strategy, based on reducing the fitness or health cost of an increasing parasite burden, has been described as naturally selected in ungulates. It is especially advantageous when resources must be allocated in confronting more severe threats [28]. Hence, male chamois may need to increase their tolerance (through raising PON1 and GPX concentrations) during the rut period, when parasite burdens increase due to the immunosuppressive influence of their reduced feeding coupled with the high testosterone levels. Males could then invest their resources in eliminating parasites or reducing their burdens, and repairing tissue lesions when the rut period is finished, their appetite returns to normal and their immune system is relieved of the aforementioned challenges. The increases in body mass that males show in summer [29] may also contribute to compensate a further cost of parasitism during the rut.

The lack of any correlation between health status and parasite infections in females could be explained by their low parasite burden at this time of year (Table 1 and [3]). Some evidence supports this hypothesis. Hypophagia displayed by males during the rutting period, especially in territorial individuals [15], would diminish the probability of introducing new infective (L3) larvae from contaminated grass. Additionally, L3 larvae of most gastrointestinal nematode species in non-naïve Caprinae take a minimum of approximately 3–4 weeks to reach adulthood [30] and, if ingested in late summer/early fall, will most likely be hypobiotic for months unless reawakened by a stressor [31]. Hence, both hypophagia and the long time required by larvae to reach adulthood, may suggest that the higher parasite richness and burden of males at that period is not only due to high testosterone concentrations, but the consequence of previously acquired infections. In fact, only one female displayed a parasite burden (2209 nematodes) close to the mean value of males (2797 nematodes). Thus, this work demonstrates that being a male has an extra physiological cost due to sex-biased parasitism that is most likely exacerbated in the autumn. This situation results from the combination of multiple parasite infections and a debilitated immune system due to malnourishment and high levels of testosterone during rut. We hypothesized that the correlation among health parameters and parasite burden is most likely initiated when tolerance to parasite infection is exceeded.

Other hypotheses should also be considered. Individual male chamois differ extensively in their energy investment in rutting activities in specific years [32]. Hence, many individuals may not be exposed to some of the detrimental effects, therefore, reducing the overall differences in mortality between males and females. Moreover, females could have their own health depletion period, such as during lactation or pregnancy, differentiated in time from the male depletion periods. This could lead males and females to be under similar stress pressures over a year-long period.

## Conclusions

Despite the moderate sample size, this study showed a sex-biased physiological cost of multiple infections by gastrointestinal and respiratory nematode parasites in a wild mammal. Interestingly, this extra-cost is mainly related to the number of different parasite species and, to a lesser extent, with the intensity of infection. The similar lifespan of females and males of this mammal suggest that the latter can deal with this cost of infection, at least during the rutting period. Further research should investigate whether this sex-biased cost of infection persists over the year and whether females suffer from these consequences of parasite infection at other times of year. On the other hand, individual-based information will be required to better understand the mechanisms driving the sex-biased parasitism, its physiological cost, and in turn, survival probabilities of this mountain ungulate.

## Abbreviations

GPX: Glutathione peroxidase
IGF1: Insulin-like growth factor
KFs: Kidney fat stores
OS: Oxidative stress
PLS: Partial least squares
PON1: Paraoxonase 1
Q^2^: Stone-Geisser test
R^2^: R squared or coefficient of determination
TAC: Total antioxidant capacity
